# Proteomic Analysis of Copper-Binding Proteins in Excess Copper-Stressed Roots of Two Rice (*Oryza sativa* L.) Varieties with Different Cu Tolerances

**DOI:** 10.1371/journal.pone.0125367

**Published:** 2015-04-28

**Authors:** Chen Chen, Yufeng Song, Kai Zhuang, Lu Li, Yan Xia, Zhenguo Shen

**Affiliations:** College of Life Sciences, Nanjing Agricultural University, Nanjing, People’s Republic of China; Zhejiang University, CHINA

## Abstract

To better understand the mechanisms involved in the heavy metal stress response and tolerance in plants, a proteomic approach was used to investigate the differences in Cu-binding protein expression in Cu-tolerant and Cu-sensitive rice varieties. Cu-binding proteins from Cu-treated rice roots were separated using a new IMAC method in which an IDA-sepharose column was applied prior to the Cu-IMAC column to remove metal ions from protein samples. More than 300 protein spots were reproducibly detected in the 2D gel. Thirty-five protein spots exhibited changes greater than 1.5-fold in intensity compared to the control. Twenty-four proteins contained one or more of nine putative metal-binding motifs reported by Smith et al., and 19 proteins (spots) contained one to three of the top six motifs reported by Kung et al. The intensities of seven protein spots were increased in the Cu-tolerant variety B1139 compared to the Cu-sensitive variety B1195 (p<0.05) and six protein spots were markedly up-regulated in B1139, but not detectable in B1195. Four protein spots were significantly up-regulated in B1139, but unchanged in B1195 under Cu stress. In contrast, two protein spots were significantly down-regulated in B1195, but unchanged in B1139. These Cu-responsive proteins included those involved in antioxidant defense and detoxification (spots 5, 16, 21, 22, 28, 29 and 33), pathogenesis (spots 5, 16, 21, 22, 28, 29 and 33), regulation of gene transcription (spots 8 and 34), amino acid synthesis (spots 8 and 34), protein synthesis, modification, transport and degradation (spots 1, 2, 4, 10, 15, 19, 30, 31, 32 and 35), cell wall synthesis (spot 14), molecular signaling (spot 3), and salt stress (spots 7, 9 and 27); together with other proteins, such as a putative glyoxylate induced protein, proteins containing dimeric alpha-beta barrel domains, and adenosine kinase-like proteins. Our results suggest that these proteins, together with related physiological processes, play an important role in the detoxification of excess Cu and in maintaining cellular homeostasis.

## Introduction

Copper (Cu) is an essential micronutrient for plant growth and development, but excess Cu is extremely toxic and interferes with numerous physiological processes, such as photosynthesis, pigment synthesis, oxidative stress, nitrogen and protein metabolism and mineral uptake. Cu binding can lead to inactivation and disturbance of protein structures [1]. Plants possess several metal-tolerance mechanisms, including compartmentation, exclusion, and chelation by organic ligands of amino acids, proteins, peptides and organic acids [1, 2]. Proteins contain cysteine (Cys), methionine (Met), and histidine residues (His), which have high affinity to divalent metal ions, and play a key role in maintaining intracellular copper homeostasis and tolerance [3, 4]. Characterizing the metal-binding proteins in plant cells is important for understanding the metal-protein interactions that may be responsible for the toxic effects of metals; metal-binding proteins act as passive molecular targets of toxic metal ions and participate in metal tolerance.

A wide range of proteins involved in Cu detoxification and homeostasis has been identified in plants [2, 5]. Identification of the metal-binding proteins involved in plant responses to heavy metal toxicity aids understanding of the molecular mechanisms of metal tolerance. Recently, immobilized metal affinity chromatography (IMAC) combined with mass spectrometry [6] has been employed to identify putative metal-binding proteins in bacterial [7], mammalian [8, 9], and plant cells [10, 11].

IMAC facilitates the separation of proteins from biological samples. It is based on specific interactions between proteins in solution and metal ions immobilized on a solid support. Metal ions are usually complexed with chelating ligands, such as iminodiacetic acid (IDA) or nitrilotriacetic acid (NTA). The proteins are separated according to their affinity for the chelated metal ions, which depends on the coordination between the chelated metal ion and electron donor groups on the protein surface. Smith et al. [9] used a Cu-IMAC approach to enrich Cu-binding proteins in hepatocellular cells. In *Streptococcus pneumoniae*, 232 and 166 putative metal-binding proteins were isolated using Cu- and Zn-IMAC columns, respectively [7]. In *Arabidopsis* roots [10] and soybean seeds [12], 35 and 32, respectively, putative Cu-binding proteins were identified. Tan et al. [11] identified 35 weak and 48 strong Cu^2+^-IMAC-interactors in *Arabidopsis* mitochondria. However, IMAC is unlikely to be suitable for specifically capturing metal-binding proteins in plants under excess metal stress conditions in which the metal-binding sites might be occupied by metal ions. Under such a condition, the proteins of interest would not interact with the immobilized metal ions and so pass through the IMAC column [10, 13].

In a previous study, we developed a new IMAC method in which an IDA-sepharose column was applied prior to the Cu-IMAC column to remove metal ions from protein samples and separate and isolate Cu-binding proteins from Cu-treated rice roots [14]. The Cu stress-induced protein expression profiles of Cu-tolerant and Cu-sensitive rice varieties were also investigated [15]. However, limited information is available on the metal-binding protein profiles in plant genotypes with different tolerances to heavy metal stress. In this study, we identified Cu-binding proteins using a Cu-IMAC column, and their respective binding motifs, in the roots of two rice varieties using 2-DE and matrix-assisted laser desorption/ionization time-of-flight/time-of-flight (MALDI-TOF/TOF) MS. The aim was to better understand the mechanisms involved in the heavy metal stress response and tolerance in plants. To our knowledge, this is the first comparative proteomic analysis of Cu-binding proteins in Cu-tolerant and Cu-sensitive crop varieties.

## Materials and Methods

### Plant growth and treatments

In this study, two varieties of rice (*Oryza sativa* L.), B1139 (Cu-tolerant) and B1195 (Cu-sensitive), initially described by Song et al. [15], were used for comparative proteomic analysis of Cu-binding proteins. Pre-germinated seeds were grown hydroponically for 7 days in Kimura B nutrient solution. The seedlings were treated with 8 μM Cu^2+^ or 0.32 μM Cu^2+^ (control) for 3 days. Three replicates were set for each treatment, and one hundred plants were cultivated for each replicates. Rice root samples were then harvested for protein extraction.

### Protein extraction and separation of Cu-binding proteins

Cu-binding proteins were extracted and separated as described previously [14]. Briefly, fresh rice roots were ground in liquid nitrogen then suspended in binding buffer (20 mM sodium phosphate, pH 5.8, 500 mM NaCl, 0.1% *w/v* Triton X-100) containing 1 mM phenylmethyl sulfonyl fluoride, incubated, and centrifuged. The proteins in the supernatant were first pre-chromatographed on a column with IDA-Sepharose to remove metal ions and then applied to the Cu-IMAC column. Copper-binding proteins were eluted with elution buffer (10 mM sodium acetate, 500 mM NaCl, pH 5.5) containing 40 mM imidazole.

### 2-DE, gel scanning and image analysis

Duplicate 2-DE gels were run three times for each treatment as described previously [14]. Briefly, for each replicate, 600 μg of copper-binding proteins were added to IPG dry strips during the rehydration step. After isoelectric focusing, gel strips were equilibrated in equilibration buffer. SDS-PAGE in the second dimension was then performed. Protein spots were visualized using a modified Coomassie brilliant blue staining method [16].

The analyses of gel images were carried out using the PDQuest software (Version 8.0; Bio-Rad). Student’s t-test was used for analysis of the difference between Cu-treated and control samples. Only spots that exhibited significant and reproducible changes between treatments were considered to be differentially expressed proteins.

### In-gel digestion and MALDI-TOF/TOF MS analysis

Selected protein spots were excised, incubated, and dehydrated for MALDI-TOF/TOF MS analysis [14, 17]. Next, peptide mass spectra were obtained using an Applied Biosystems 4700 Proteomics Analyzer MALDI-TOF/TOF mass spectrometer (Applied Bio-systems, Framingham, MA). All spectra of proteins were searched against the NCBInr (National Center for Biotechnology nonredundant database) using online MASCOT (http://www.matrixscience.com). In these searches the peptide mass tolerance was set at 0.15 Da, mass tolerance of TOF—TOF fragments was set to 0.25 Da, one missed cleavage by trypsin was allowed, and carbamidomethyl of Cys as fixed modification, and oxidation of Met as variable modification were included. Only significant hits, as defined by the MASCOT probability analysis (P<0.05), were accepted.

## Results

### Separation and 2-DE analysis of Cu-IMAC-binding proteins

Rice seedlings were exposed to Kimura B nutrient solution containing 8 μM Cu^2+^ for 3 days, while control plants were grown in normal nutrient solution containing 0.32 μM Cu. Rice root proteins were extracted with binding buffer containing 1 mM phenylmethylsulfonyl fluoride (PMSF). A 24-mg protein sample was pre-chromatographed on a column with IDA-Sepharose before being applied to a Cu-IMAC column for separation of Cu-binding proteins. Following washing with nine bed volumes of washing buffer containing 10 mM imidazole, non-specifically bound proteins were removed from the column. Cu-binding proteins were obtained following elution with elution buffer containing 40 mM imidazole (Table 1). The yields of copper-binding proteins from the roots of the two rice varieties treated with 8 μM Cu^2+^ were markedly higher than yields from control plants, indicating that Cu treatment increased the amount of proteins specifically bound with the Cu-IMAC. The yield of Cu-IMAC-binding proteins from the roots of the Cu-tolerant rice variety B1139 than was higher than that from the Cu-sensitive variety B1195.

**Table 1:** Effect of copper stress on yield of copper-binding proteins in the rice roots of varieties B1139 and B1195.

Rice varieties	Copper-binding proteins yield(μg/mg protein)		% of control
	Control(0.32 μmol/Cu^2+^)	8 μmol/Cu^2+^	
B1139	30.16±0.33	40.72±0.22	135.02 a
B1195	30.78±0.39	38.61±0.12	125.46 b

Note: The copper-binding protein yield is expressed in terms of micrograms copper-binding proteins per milligrams total proteins. The letters of a and b about change of copper-binding proteins yield under copper stress indicate a statistically significant difference (*P<0*.*05*) between two varieties by Duncan’s test.

**Fig 1:**
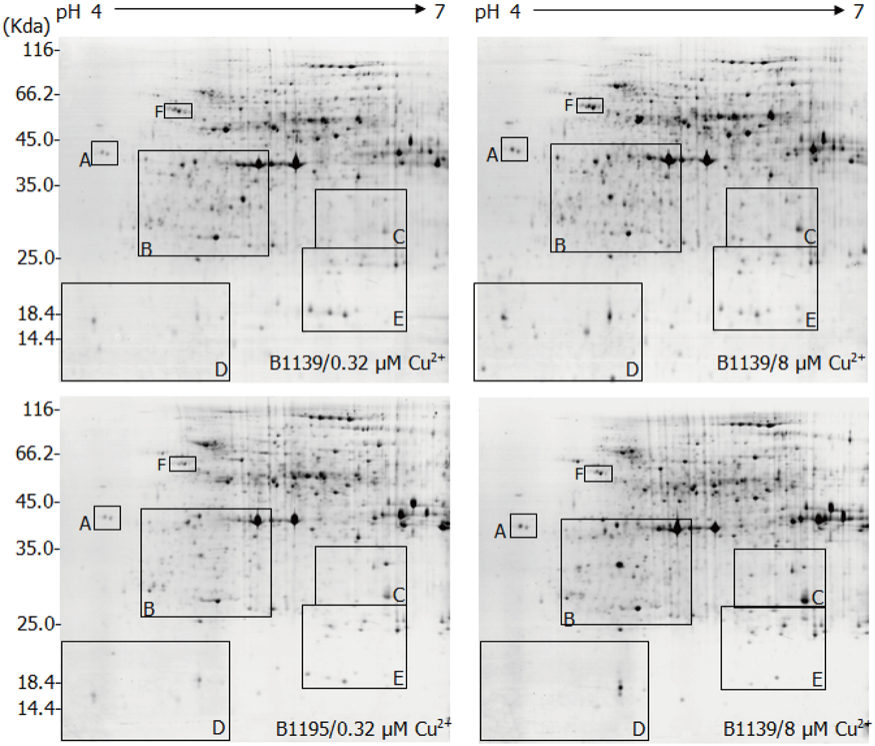
Representative 2-DE maps of the rice B1139 and B1195 roots copper-binding proteins eluted from a Cu-IMAC column with elution buffer containing 40 mmol imidazole. The 7 days seedlings were treated with 8 μmol Cu^2+^ for 3 days. Seedlings without any treatment, grown in a full-strength nutrient solution were used as a control (0.32 μmol Cu^2+^). 100 μg of copper-binding protein were loaded onto IPG dry strips (17 cm, pH 4–7 linear gradient; Bio-Rad) during the rehydration step (13 h), followed by focusing for a total of 6,000 V·h using a PROTEAN IEF CELL (Bio-Rad). SDS-PAGE in the second dimension was carried out using 12% SDS—polyacrylamide gels. The protein spots were visualized by mass spectrometry-compatible silver staining.

**Fig 2:**
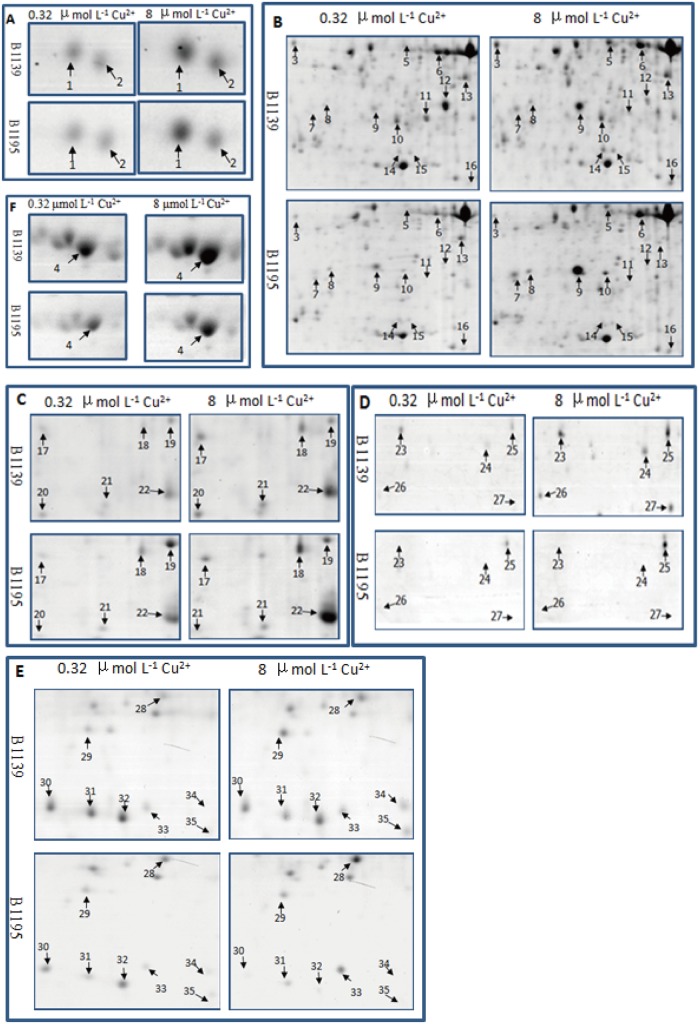
Enlargements of the framed areas shown in Fig 1. The framed regions A-E in Fig 1 are enlarged and compared in detail. The arrows indicate the distinct copper-binding proteins between rice B1139 and B1195 roots.

Copper-binding protein maps produced from 2-DE gels showed high reproducibility among the three independent extractions. Fig 1 shows representative gels of Cu-IMAC-binding proteins extracted from control and Cu-treated roots. Approximately 320 protein spots were reproducibly detected on silver-stained gels using the PDQuest 8.0 software. Six typical regions are enlarged in Fig 2. Quantitative image analysis revealed that a total of 35 protein spots exhibited more than a 1.5-fold change in intensity between the control and copper treated samples in at least one variety. Among these, the expression of six protein spots showed no significant difference between the two rice varieties under Cu stress. Compared to the control, the 8 μM Cu^2+^ treatment resulted in increases in the intensity of 17 protein spots and decreases in 3 spots (spots 30, 31 and 32) in both varieties. Of these 17 up-regulated protein spots, 7 (spots 5, 8, 10, 16, 18, 21 and 25) were increased in the Cu-tolerant variety B1139 compared to the Cu-sensitive variety B1195 (*p*<0.05). Six spots were markedly up-regulated (spots 23 and 24) or newly induced (spots 26, 27, 34 and 35) in B1139, but not detected in B1195 (*p*<0.05). Four spots (spots 3, 14, 15 and 29) were significantly up-regulated in B1139, but unchanged in B1195 under Cu stress. In contrast, protein spot 33 was up-regulated and spots 13 and 19 were down-regulated in B1195, but were unchanged in B1139 under Cu stress. Spots 11 and 12 were down-regulated in B1139, but not detected in B1.

### Identification of Cu-IMAC-binding proteins

The 34 differentially expressed protein spots were identified by MALDI-TOF/TOF MS. Table 2 shows the identity of 27 Cu-binding proteins after a database search. These Cu-responsive proteins included those involved in antioxidant defense and detoxification (spots 5, 16, 21, 22, 28, 29 and 33), pathogenesis (spots 23 and 26, 24 and 25), regulation of gene transcription (spots 8 and 34), amino acid synthesis (spot 6 and 11 and 12), protein synthesis, modification, transport and degradation (spots 1 and 2, 4, 10, 15, 19, 30, 31, 32 and 35), cell wall synthesis (spot 14), molecular signaling (spot 3), and salt stress (spots 7, 27 and 9); together with other proteins, such as a putative glyoxylate induced protein (spot 18), proteins containing dimeric alpha-beta barrel domains (spot 20), and adenosine kinase-like proteins (spot 13).

**Table 2:** Differentially expressed copper-binding proteins identified by MS/MS.

Spot no.^a^ ^)^	Fold-ch.^b^ ^)^ 39/95	NCBI Acc.no.^c^ ^)^	Protein name	Mr/PI		SC^d^ ^)^ (%)	Score	PM^e^ ^)^	MS/MS peptide sequence >95% C.I. (Indv. ion score)
				Theoretical	Observed				
**Proteins involved in antioxidation and detoxification**									
5	4.41/3.88 a↑/b↑	AAG46133	Putative peroxidase	35.4/7.55	42.1/5.16	5	130	1(1)	GLDAEDMVVLSGAHTVGR(130)
22	2.4/3.2 b↑/a↑	AAC04837	Germin-like protein 6	24.5/5.92	26.6/6.54	28	388	6(5)	AAMLDTPR(42); YVNADHFFK(57); HSPVLVNGFACLDPK (98); IDYAPLGENPPHTHPR(120); GTIDWLQAQFWENNHY(71)
28	2.5/2.1 a↑/a↑	BAB92583	Putative quinone-oxidoreductase(QR2)	21.7/6.06	23.3/6.36	48	392	6(6)	VYVVYYSMYGHVAK(74); IWQVPETLHEEVLGK(83); WP TEMELEHAFHQGK(36); MFNMGEVQGGSPYGAGTFAAD GSR(109); MGAPPKPDVPTITPQELTEADGILFGFPTR(90)
29	2.7/1.1 a↑/b-	Q7XUP7	Methionine sulfoxide reductase A2-1	21.1/5.88	21.5/5.99	35	472	8(5)	HNPTTLNR(55); IVTEILPATR(62); TEVGYSQGHR(77); FYPAEEYHQR(65); DVCGGGTGHAEVVR(137)
33	1.2/4.6 b↑/a↑	AAA33917	Copper/zinc superoxide dismutase	15.3/5.71	17.7/6.29	15	175	2(2)	EHGAPEDETR(76); AVVVHADPDDLGK(99)
16	3.8/1.8 a↑/b↑	AAC64007	Glutathione S-transferase II	24.3/5.77	24.8/5.47	37	479	6(6)	VVEENLEK(65); VLEVYEAR(71); GEHKAPDHLAR(60); LYGSTLSWNVTR(91); NPFGQVPALQDGDLFLWESR(90); CVAVLEEAGAEYEIVPLDFSK(101)
21	3.3/1.5 a↑/b↑	AAC64007	Glutathione S-transferase II	24.3/5.77	26/6.3	27	358	5(5)	VVEENLEK(65); VLEVYEAR(75); GEHKAPDHLAR(50); LYGSTLSWNVTR(100); CVAVLEEAGAEYEIVPLDFSK(68)
**Proteins involved in transcriptional regulation**									
8	2.32/1.72 a↑/b↑	BAC65369	CHP-rich zinc finger protein-like	28.4/4.95	32.9/4.75	23	326	5(4)	DHDMKER(41); KMEDDFDAFTASK(75); IAIAVDLSDESA YAVR(117); LGSVSDYCVHHCVCPVVVVR(71);
34	new/none	BAC66711	Putative cold shock protein-1	19.0/6.28	17.9/6.51	74	704	8(7)	GYGGGGGGYGGGDR(87); GYGGGGGYGGGGGGGS R(107); DCSQGGGGGGGYGGGGGGYR(136); SLNDGDVV EFSVGSGNDGR(138); GGGGGGGGGGCYNCGETGHI AR(102); AVDVTAPGGGALTGGSRPSGGGDR(45); GFGF ITPDDGGEDLFVHQSSLK(62)
**Proteins involved in protein synthesis, modification, transport and degrdation**									
30	0.57/0.01 a↓/b↓	ABA98689	Putative eukaryotic translation initiation factor 5A-2	17.8/5.6	17.6/5.9	45	364	5(5)	TYPQQAGTIR(59); LPTDDNLLSQIK(99); CHFVAIDIFT AK(102); DLVVTVMSAMGEEQICALK(38); KLEDIVPSS HNCDVPHVNR(68)
31	0.14/0.6 b↓/a↓	ABF98987	Putative eukaryotic translation initiation factor 5A-2	17.9/5.87	17.0/6.0	40	270	6(4)	NGHIVIK(37); TYPQQAGTIR(62); CHFVAIDIFNGK(79); DDLRLPSDEALLTQIK(43);
32	0.57/0.02 a↓/b↓	AAC67555	Translation initiation factor 5A	17.7/5.77	16.8/6.2	35	479	6(6)	TYPQQAGTIR(57); LPTDDSLLGQIK(84); CHFVAIDIFN GK(92); DDLRLPTDDSLLGQIK(54); LEDIVPSSHNCDVPHV NR(94); KLEDIVPSSHNCDVPHVNR(100)
19	0.89/0.47 a-/b↓	BAD26337	Putative elongation factor EF-2	94.9/5.85	42.2/6.55	10	423	7(6)	GGGQVIPTAR(41); VIYASQLTAKPR(73); RVIYASQLTAKPR(56); ILSEEFGWDKDLAK(81); AYLPVIESFGFSSQLR(103); GHVFEEMQRPGTPLYNIK(46)
4	1.57/1.52 a↑/a↑	AAX85991	Protein disulfide isomerase	57.05/4.95	71.5/4.9	18	466	8(7)	NIQEYKGPR(40); GDAAVERPLVR(37); TADEIVDFIK K(64); VVVADNVHDFVFK(104); AHVEPDQIVSWLK(88); SDYDFGHTLHANHLPR(50); VVTFDKNPDNHPYLLK(56)
35	new/none	ABA99827	Putative ubiquitin-conjugating enzyme spm2	16.8/6.42	16.5/6.52	46	292	6(4)	FSLLSNWR(39); EYTMEAILTQLKK(81); LFCDKDYPDRPPTVK(53); SWTGTIIGPHNTVHEGR(59)
15	1.7/1.1 a↑/b-	BAA94966	Epsilon-COP 1	31.7/5.23	27.5/5.18	32	551	6(6)	AVSAEDNFER(68); LSHPDHVLVK(48); EAYLIFQDFAE K(94); EWLSDSAVGSNPVLR(119); LIAGIIFMHEQDYTEA LK(112); NLFYLGAYQAAINNSDVPGLDADAAAER(109)
10	3.23/2.23 a↑/b↑	AAX11351	Cathepsin B-like cysteine protease	40.4/6.25	30.9/5.11	23	550	8(8)	HFSVNAYR(66); GWGDDGYFK(62); KHFSVNAYR(61); HITGGMMGGHAVK(78); PGCEPAYPTPVCEK(90); GTNEC GIEEDVVAGMPSTK(99); GVVTDECDPYFDQVGCK(93)
1	2.21/2.34 a↑/a↑	P25776	Oryzain alpha chain; Flags: Precursor	51.3/5.14	43.1/4.2	15	563	6(6)	SWGESGYVR(61); CGIAVEPSYPLK(71); AFQLYSSGIFTGK (102); CGIAVEPSYPLKK(70); AVANQPVSVAIEAGGR(99); CGTALDHGVAAVGYGTENGK(159)
2	2.28/2.07 a↑/a↑	P25776	Oryzain alpha chain; Flags: Precursor	51.3/5.14	42.1/4.24	8	222	3(3)	SWGESGYVR(42); AFQLYSSGIFTGK(88); AVANQPVSVAIEAGGR(93)
**Pathogenesis-related protein**									
23	1.8/none a↑/	BAC56823	Putative pathogenesis- related protein	18.2/4.76	19.6/4.25	20	220	3(3)	STTSIGCAR(37); ADYVYSSNTCTR(72); GALLDCGH YTQVVWR(111)
26	new/none	BAC56830	putative pathogenesis-related protein	19.4/4.37	11.7/4	41	216	4(3)	AGDCALIHSGSWEK(94); RVEGVGEVVWDDAVAA YAENYAAER(37); VDCDNGGVFITCNYNPAGNFQG ERPFER(54)
25	7.5/2.3 a↑/b↑	ABA99548	Pathogenesis-related protein Bet v I family protein	17.2/4.96	16.9/4.99	63	711	7(7)	AVAVSVER(55); APAFVSDER(94); VCLDVHSLPK(69) IVVCDSATHVLK(76); SHSTETKLEATGDATCVAK (158); LTVEYELEDGASLSPEQEK(149); VCAGFIDA VEVEGNGGPGTIHIMK(109)
24	17.3/none a↑/	AAF85972	Pathogenesis-related protein PR-10a	16.9/4.95	14.2/4.81	18	165	2(2)	VAVCDAASHVLK(55); APACVSDEHAVAVSAER(109)
**Proteins involved in amino acid synthesis**									
6	2.35/5.81 b↑/a↑	P14656	Glutamine synthetase shoot isozyme	39.4/5.51	40.8/5.32	13	407	5(5)	DIVDSHYK(60); NDGGYEIIK(74); EHISAYGEGNE R(70); HKEHISAYGEGNER(104); HETADINTFSWG VANR(99);
11	0.34/none a↓/	P14656	Glutamine synthetase shoot isozyme	39.4/5.51	31.5/5.27	13	250	5(5)	DIVDSHYK(67); NDGGYEIIK(72); EHISAYGEGN ER(91); HKEHISAYGEGNER(105); IIAEYIWIGGSG MDLR(49)
12	0.13/none a↓/	AAN05339	Putative glutamine synthetase root isozyme	38.8/5.73	33/5.36	17	456	6(5)	DIVDAHYK(63); GPITDVSQLPK(81); EHIAAYGEGNE R(86); HKEHIAAYGEGNER(103); IIAEYIWVGGSGID LR(109)
**Protein involved in cell wall synthesis**									
14	2.3/1.1 a↑/b-	BAA81774	Putative caffeoyl-CoA O-methyltransferase 1	27.9/5.11	27.5/5.13	38	570	8(6)	YVLDTTVLPR(59); YHEQLLQLVR(74); ESYEIGRPFL EK(51); GLEKLDELLAEEAAAGR(112); IDVCQLAIAD GITICR(119); EAAFDFAFVDADKPNYVK(108)
**Protein involved in signal molecular**									
3	2.20/0.67 a↑/b-	BAA88900	Calcium-binding protein	48.07/4.52	40.3/4.56	18	469	7(6)	KVHTIFTK(56); NDKNHLIK(49); TLVLQFSVK(43); HEQKLDCGGGYVK(118); QSGSIYEHWDILPPK(89); FYAISAEYPEFSNKDK(91)
**Proteins induced by salt stress**									
7	5.32/5.22 a↑/a↑	AAB53810	Salt gene product	15.2/5.0	31.8/4.67	45	517	5(5)	LLGVTIYSSDAIR(90); SGTLIDAIGIYVHP(106); KLL GVTIYSSDAIR(110); EFSIPLQDSGHVVGFFGR(84); EISGTHGPVYDLADIVTYLK(126);
27	new/none	AAB53810	Salt gene product	15.2/5	9.2/5.04	51	506	6(5)	LLGVTIYSSDAIR(102); SGTLIDAIGIYVHP(108); KLL GVTIYSSDAIR(93); EFSIPLQDSGHVVGFFGR(92); EISGTHGPVYDLADIVTYLK(103)
9	5.61/5.17 a↑/a↑	A2WPN7	Salt stress-induced protein	15.2/5	33.1/5	66	656	6(6)	LLGVTIYSSDAIR(118); SGTLIDAIGIYVHP(108); KLL GVTIYSSDAIR(113); EFSIPLQDSGHVVGFFGR(118); EISGTHGPVYDLADIVTYLK(121); SIAFNYIGVDGQ EYAIGPWGGGEGTSTEIK(79)
**The other proteins**									
18	2.9/2.1 a↑/b↑	BAC83197	Putative glyoxylate induced protein	34.2/5.98	31.1/6.43	30	515	7(7)	DVNRDPLI(42); QPLLETPGEVFELR(102); HSVISDE VTTLVIFER(132); YTTIEGYHPDLIVGSTDK(44); A TNPTLAPAHLQDLPGFTR(68); RYTTIEGYHPDLIVG STDK(36); EVHYNQHGLLLLEGQGIYR(90)
20	2.8/3.3 b↑/a↑	NP_001060247	Protein containing dimeric alpha-beta barrel domain	26.5/7.11	25.5/6.01	16	373	4(4)	VSFGENFSPAR(82); LKEGVEAHQLAEK(89); SPAA EALGPTHVLHSR(117); LRSPAAEALGPTHVLHSR(85)
13	1.1/0.21 a-/b↓	AAO72629	Adenosine kinase-like protein	40.6/5.57	37/5.45	35	780	8(8)	KPENWALVEK(79); HLPMYDELASK(66); AGCYAAN VIIQR(82); GNVEYIAGGATQNSIR(155); VRGWETE NVEEIALK(76); VLPFVDYIFGNETEAR(115); IAVIT QGADPVVVAEDGQVK(128); NAQAAGVTAHYYEDE AAPTGTCAVCVVGGER(79)

Note: Differentially expressed copper-binding proteins identified by MS/MS.;

^a^ Spot no., numbering corresponds to the 2-DE gel;

^b^ Fold-ch., Fold-changes, the fold-changes of each spot was calculated by %Vol in treated samples/%Vol in control samples;

^↑^, up-regulated;

^↓^, down-regulated;

-, no change;

^c^ Acc. no., accession number in NCBI database;

^d^ SC, sequence coverage by MS/MS;

^e^ PM, number of peptides matched;

The a and b of letter about fold-changes indicate a statistically significant difference (p≤0.05) between B1139 and B1195 by Duncan’s test.

### Analysis of metal-binding motifs

Metals often bind proteins at specific coordination sites involving Cys, His, and Met residues [18]. Smith et al. [9] reported nine putative metal-binding domains, H-(X)n-H (n = 0–5) and C-(X)n-C (n = 2–4). In *Arabidopsis* roots, Kung et al. [10] found that 29 of 35 identified Cu-binding proteins possessed one or more of the H-(X)_n_-H (n = 0–5) and C-(X)_m_-C (n = 2–4) metal-binding motifs suggested by Smith et al. [9]. Kung et al. [10] further described the top six candidate motifs (H-(X)_5_-H, H-(X)_7_-H, H-(X)_12_-H, H-(X)_6_-M, M-(X)_7_-H and H-(X)_3_-C), which were found in 31 of 35 proteins (89%). In this study, 27 proteins (in 34 protein spots) were confidently identified. Among these 27 proteins, 22 contained one or more of the nine putative metal-binding motifs reported by Smith et al. [9] (Table 3), while 17 contained one to three of the top six motifs reported by Kung et al. [10] in rice roots. Fourteen proteins contained both the motifs reported by Smith et al. [9] and the top six motifs reported by Kung et al. [10] Three proteins (calcium-binding protein (CBP), putative caffeoyl-CoA O-methyltransferase 1, and a salt gene product), contained neither the motifs reported by Smith et al. [9] nor the top six motifs reported by Kung et al. [10]. CBP and CCoAOMT 1 respectively contained 8 (H-(X)_6_-H, H-(X)_9_-H, H-(X)_5_-C, H-(X)_7_-C, C-(X)_6_-H, C-(X)_7_-H, M-(X)_5_-H and M-(X)_5_-C) and 7 (H-(X)_11_-H, M-(X)_3_-M, M-(X)_11_-M, C-(X)_10_-H, C-(X)_0_-M, H-(X)_4_-M and M-(X)_9_-H) of the 117 potential metal-binding motifs. Among these motifs, H-(X)_9_-M, M-(X)_5_-H, M-(X)_3_-M, M-(X)_11_-M and H-(X)_4_-M are thought to be enriched motifs in Cu-IMAC proteins [10].

**Table 3:** Potential Cu-binding motifs of identified proteins.

Spot no.	Protein name	Reported motif I^a^	Reported motif II^b^
1	Oryzain alpha chain; Flags: Precursor	C-(X)_2_-C; C-(X)_4_-C; H-(X)_5_-H	H-(X)_5_-H; H-(X)_3_-C
2	Oryzain alpha chain; Flags: Precursor	C-(X)_2_-C; C-(X)_4_-C; H-(X)_5_-H	H-(X)_5_-H; H-(X)_3_-C
3	Calcium-binding protein	–	–
4	Protein disulfide isomerase	C-(X)_2_-C; H-(X)_2_-H; H-(X)_5_-H	H-(X)_5_-H
5	Putative peroxidase	C-(X)_4_-C; H-(X)_5_-H	H-(X)_5_-H
6	Glutamine synthetase shoot isozyme	H-(X)_2_-H	H-(X)_3_-C
7	Salt gene product	–	–
8	CHP-rich zinc finger protein-like	C-(X)_3_-C; H-X_0_-H; H-(X)_4_-H; H-(X)_5_-H	H-(X)_5_-H; H-(X)_6_-M; H-(X)_3_-C
9	Salt stress-induced protein	–	H-(X)_7_-H
10	Cathepsin B-like cysteine protease	C-(X)_2_-C; C-(X)_3_-C; C-(X)_4_-C	H-(X)_7_-H; H-(X)_3_-C; H-(X)_12_-H
11	Glutamine synthetase shoot isozyme	H-(X)_2_-H	H-(X)_3_-C
12	Putative glutamine synthetase root isozyme	H-(X)_2_-H	H-(X)_7_-H; H-(X)_3_-C
13	Adenosine kinase-like protein	C-(X)_2_-C	–
14	Putative caffeoyl-CoA O-methyltransferase 1	–	–
15	Epsilon-COP 1	H-(X)_1_-H; H-(X)_2_-H;	H-(X)_7_-H
16	Glutathione S-transferase II	C-(X)_3_-C; H-(X)_4_-H	–
18	Putative glyoxylate induced protein	H-(X)_3_-H	H-(X)_7_-H
19	Putative elongation factor EF-2	C-(X)_3_-C; C-(X)_4_-C; H-(X)_2_-H; H-(X)_4_-H	M-(X)_7_-H;H-(X)_3_-C
20	Protein containing dimeric alpha-beta barrel domain	H-(X)_2_-H	–
21	Glutathione S-transferase II	C-(X)_3_-C; H-(X)_4_-H	–
22	Germin-like protein 6	H-(X)_1_-H	H-(X)_7_-H
23	Putative pathogenesis-related protein	C-(X)_4_-C	–
24	Pathogenesis-related protein PR-10a	–	M-(X)_7_-H
25	Pathogenesis-related protein Bet v I family protein	–	M-(X)_7_-H
26	putative pathogenesis-related protein	C-(X)_4_-C	–
27	Salt gene product	–	–
28	Putative quinone-oxidoreductase(QR2)	H-(X)_0_-H; H-(X)_2_-H;	H-(X)_6_-M
29	Methionine sulfoxide reductase A2-1	H-(X)_1_-H	–
30	Putative eukaryotic translation initiation factor 5A-2	H-(X)_0_-H; H-(X)_1_-H; H-(X)_3_-H; H-(X)_5_-H	H-(X)_5_-H
31	Putative eukaryotic translation initiation factor 5A-2	H-(X)_0_-H; H-(X)_1_-H; H-(X)_3_-H; H-(X)_5_-H	H-(X)_5_-H
32	Translation initiation factor 5A	H-(X)_0_-H; H-(X)_1_-H; H-(X)_3_-H; H-(X)_5_-H	H-(X)_5_-H
33	Copper/zinc superoxide dismutase	H-(X)_1_-H; H-(X)_2_-H; H-(X)_4_-H	H-(X)_7_-H
34	Putative cold shock protein-1	C-(X)_2_-C	–
35	Putative ubiquitin-conjugating enzyme spm2	H-(X)_3_-H	–

Note:—indicates not present; X represents any amino acid, C represents cysteine, H represents histidine, M represents methionine;

^a^ Motifs that were reported by Smith et al.;

^b^ Motifs that were reported by Kung et al

## Discussion

In our previous study, the Cu-IMAC plus IDA-Sepharose pre-chromatography method was used for the separation and isolation of Cu-binding proteins extracted from the roots of rice seedlings exposed to excess Cu, and six novel Cu-binding proteins (peroxidase, quinone-oxidoreductase QR2, epsilon1-COP, NADPH-dependent mannose 6-phosphate reductase, cytidine/deoxycytidine deaminase, and caffeoyl-CoA O-methyltransferase 1) were identified [14]. In this study, 26 Cu-binding proteins were found to be differentially expressed in Cu-stressed rice roots. Of these proteins, 10 (elongation factor EF-2, glutamine synthetase, calcium-binding protein, adenosine kinase, glutathione S-transferase, peroxodase, quinone-oxidoreductase QR2, protein disulfide isomerase, epsilon-COP1 and caffeoyl-CoA *O*-methyltransferase 1) were identified as Cu-IMAC-binding proteins in Arabidopsis [10, 11], soybeans [12] and rice [14]. However, to our knowledge, the other 16 proteins (spots 1, 2, 7, 27, 8, 9, 10, 18, 20, 22, 23, 26, 24, 25, 29, 30, 31, 32, 33, 34 and 35) have not been reported as Cu-IMAC-binding proteins in plants or animals. Further studies are required to clarify their roles in plant cells.

In this study, six proteins involved in antioxidant defense and detoxification were identified as Cu-IMAC-binding proteins: a putative peroxidase (spot 5), germin-like protein 6 (spot 22), a putative quinone-oxidoreductase (QR2) (spot 28), methionine sulfoxide reductase A2-1 (spot 29), copper/zinc superoxide dismutase (spot 33) and glutathione S-transferase II (spots 16 and 21). Significant up-regulation of these detected Cu-binding proteins was observed in both rice varieties. Plant responses to Cu-induced oxidative stress are often mediated through antioxidant defense systems, which include enzymatic components, such as superoxide dismutase (SOD), catalase (CAT), peroxidase (POD), ascorbate peroxidase (APx), dehydroascorbate reductase and glutathione reductase (DHAR). Song et al. [15] reported that the up-regulation of POD, APX and DHAR was more pronounced in the Cu-tolerant variety B1139 than in the Cu-sensitive variety B1195. The up-regulation of another APX was observed only in the Cu-tolerant variety B1139. It was suggested that the B1139 variety has a greater ability to scavenge H_2_O_2_ than B1195 under Cu stress. In this study, the up-regulation of four proteins (putative peroxidase, putative quinone-oxidoreductase, methionine sulfoxide reductase A2-1 and glutathione S-transferase II) was also more pronounced in the B1139 variety than in B1195. In contrast, the up-regulation of CuZn-SOD (spot 33) and germin-like protein 6 (spot 22) was more pronounced in the Cu-sensitive variety B1195 than in the Cu-tolerant variety B1139. SODs, which catalyze the dismutation of O_2_^•-^ to H_2_O_2_ and O_2_, are metalloenzymes that occur in three molecular forms containing manganese (Mn-SOD), iron (Fe-SOD) or copper and zinc (CuZn-SOD) as prosthetic metals.

Germin-like proteins (GLPs) are ubiquitous plant glycoproteins belonging to the cupin super family, which play important roles in plant development and plant defense responses. Some GLPs possess SOD activity and each GLP contains two amino acid sequence motifs [19, 20]. Six germin proteins (which each bind a single manganese ion) comprise a stable hexamer structure. The omission of Mn^2+^ from GLP4 in barley resulted in the loss of SOD activity [20]. Overexpression of OsGLP1 in tobacco led to the hyper-accumulation of H_2_O_2_ and reinforcement of the cell wall components and increased tolerance against biotic and abiotic stresses [21]. SOD and GLP lead to the production of H_2_O_2_. Li et al. [22] reported that 100 μM Cu decreased the expression of a GLP subfamily 3 member precursor in the Cu-tolerant plant *Elsholtzia splendens*.

Proteomic analyses of proteins responsive to ion toxicity in several plant species also showed that expression of CuZn-SOD was stimulated by Cd and Al [23, 24]. Increased expression of certain GLPs or genes was also observed in various plants under drought, salt, Al and heavy metal stresses (Cd^2+^, Cu^2+^ and Co^2+^), and pathogen invasion [19, 25, 26]. However, to our knowledge, no proteomics-based study on metal-induced alterations of CuZn-SOD and GLPs as Cu-IMAC-binding proteins in plants has been published.

Protein spot 28 was identified as quinone oxidoreductase 2 (QR2) and its intensity was markedly increased by Cu stress. In plants, quinones are redox-active compounds that can oxidize the thiol groups of proteins and glutathione. Quinone redox changes are catalyzed by QRs, a subfamily of medium-chain dehydrogenase/reductases. At least two major types of QR exist in plants: the ζ-crystallin-like QR1s, which catalyze single electron reductions of quinones to semiquinone radicals, and the DT-diaphorase like QR2s, which catalyze two electron reductions of quinones to hydroquinones. Up-regulation of the QR2 protein may contribute to preventing excessive oxidative damage in plants under stress. The expression level of QR increases in *Capsicum annuum* leaves in response to herbivore attack [27] and in tomato roots to Al stress [28]. In contrast, Li et al. [22] reported that 100 μM Cu decreased the expression of a quinone oxidoreductase-like protein in *Elsholtzia splendens* roots.

It was suggested that GSTs play a role in stress-response pathways in rice [29]. A major function of GSTs is to detoxify a variety of hydrophobic, electrophilic compounds by catalyzing their conjugation with GSH. Biochemical studies have shown that Cu ions interact with GSTs by directly binding to the peptide [10, 30]. In this study, spots 16 and 21 were identified as GST II and were up-regulated in rice roots exposed to Cu. GSTs were up-regulated by Cu [15, 31], Zn [32], Al [33] and osmotic stress [34] in rice, as well as in other plant species exposed to Cu [35] or Cd [36]. Whether GST proteins are involved in Cu homeostasis or stress through direct binding remains to be determined.

One of the identified proteins (spot 29) was methionine sulfoxide reductase A2-1 (MsrA2-1). Quantitative analysis showed that Cu treatment significantly increased the levels of this protein in the Cu-tolerant variety B1139 and had no significant effect in the Cu-sensitive variety B1195. Under a range of environmental stresses, ROS oxidize Met to two diastereoisomers of Met sulfoxide (MetO), *S*-MetO and *R*-MetO, depending on the position of the oxygen atom on sulfur. This amino acid conversion could lead to changes in the activity and conformation of proteins [37]. *S*-MetO and *R*-MetO are reduced back to Met by MsrA and MsrB, respectively. Thus, Msrs are known to play an important role in the response of plants to environmental stresses [38, 39]. Differential expression of MsrA2 was observed in *Chlamydomonas reinhardtii* cells under Mn deficiency [40]. MsrA2, encoding a cytosolic isoform of the enzyme, is presumed to repair oxidized proteins in the dark, thus preventing cellular oxidative damage [41]. The overexpression of plastidial *MsrA4* (*PMSR4*) in Arabidopsis confers resistance to methyl viologen (MV)-induced oxidative damage, whereas knockout leads to susceptibility [42].

Four of the identified protein spots were putative pathogenesis-related (PR) proteins (spots 23 and 26), PR-10a (spot 24) and pathogenesis-related Bet v 1 family proteins (spot 25). Bet v 1 is a member of the PR-10 multigene family. Bet (spot 25) was up-regulated in B1139 compared to B1195, as were the expressions of other PRs (spot 26, 23 and 24). The up-regulation of PR proteins has also been observed in other plant species exposed to heavy metal stress (Cd, Cu and Zn) [22, 31, 36, 43]. PR proteins participate in a wide range of cell functions, including cell wall rigidity, signal transduction, and antimicrobial activity. Overexpression of PR proteins in plants or cells increased tolerance to salt, osmotic [44, 45], cold [46], heavy metal and pathogen stresses [47].

In this study, spot 34 was identified as a putative cold-shock domain protein (*Oryza sativa* CSD protein); its homolog has been reported as OsCSP2. This protein was detected only in the Cu-treated B1139 variety. Cold-shock proteins (CSPs) function as RNA chaperones by destabilizing the RNA secondary structure and promoting translation and transcription [48], and play a critical role in the adaptation of bacteria to low temperatures. CSPs contain a specialized DNA/RNA binding domain known as a cold-shock domain (CSD). The CSD includes two consensus RNA-binding motifs (RNP1 and RNP2). In rice, OsCSP1 and OsCSP2 contain an N-terminal CSD and glycine-rich regions that are interspersed by four and two CX2CX4HX4C (CCHC) retroviral-like zinc fingers, respectively. “Zinc-finger” refers to a type of protein domain in which a zinc atom is surrounded by Cys and/or His residues. The zinc finger proteins form a relatively large family of transcription regulators in plants and play important roles in plant development and response to environmental stress. It is possible that Cu ions are bound by the His or Cys of the zinc-finger domain under excess Cu conditions. Further studies are required to fully characterize the changes in the function of zinc finger proteins due to Cu binding.

The intensity of protein spot 8, identified as a CHP-rich zinc finger protein-like (CHP), was significantly increased by Cu stress in both rice varieties. This protein is rich in Cys, His, and Pro, with three divergent C1 (DC1) domains and the capacity to bind two zinc ions. DC1 domain-containing proteins have been implicated in plant responses to abiotic stress [49]. The up-regulation of CHP proteins has also been observed in cold-stressed Arabidopsis [50] and *Xanthomonas oryzae*-inoculated rice [51]. However, it was reported that CHP gene expression levels are reduced by salt or drought stress, as well as by exogenous ABA supply [52] and *Pseudomonas fluorescens* infection [53]. The expression of *TaCHP* in a salt-hypersensitive wheat cultivar and in Arabidopsis enhanced their tolerance to salt stress.

The abundances of several proteins involved in amino acid and protein synthesis, protein modification, and intracellular transport and degradation were altered in this study. Three protein spots were identified as glutamine synthetase [54] shoot isozyme (spots 6 and 11) and root isozyme (spot 12). Protein spot 6 was upregulated in both rice varieties. Spots 11 and 12 were down-regulated in B1139 and not detected in B1195. GS is a key enzyme in NH_4_^+^ assimilation and catalyzes the ATP-dependent fixation of the δ-carboxyl group of glutamate to form glutamine. Plant GS binds two magnesium ions at specific metal-binding sites in each subunit for activity [55]. The activity of GS can be affected by heavy metals. Decreased GS activity and expression were shown in plants exposed to excess Cu, Cr, Ni and Cd [56–59]. It was also reported that GS in plants is particularly prone to proteolysis under oxidative stress conditions [60]. Excess Cu was demonstrated to induce ROS production and to increase the expression of proteinases, such as CBCP (spot 10) and oryzain (spots 1and 2) [54]. In contrast, the up-regulation of GS proteins has also been observed in plants or germinating seeds subjected to cold, salt, Cu and Cd stress [61–63]. Further studies are necessary to elucidate the effects of Cu binding on GS protein regulation and function.

The eukaryotic translation initiation factor 5A (eIF5A) is a small protein ubiquitously present in eukaryotic cells and contains the unusual amino acid hypusine. eIF5A was also suggested to play a role in translation elongation [64] and other aspects of RNA metabolism, such as RNA export [65], as a translation initiation factor. The expression of eIF5A in plants usually increases in response to abiotic stress [66, 67]. Transgenic Arabidopsis plants overexpressing *RceIF5A* exhibited increased resistance to heat, oxidative and osmotic stresses, while plants with reduced expression are more susceptible to these stresses [67]. Chou et al. [68] found two cDNA clones (OseIF5A-1 and OseIF5A-2) encoding eIF5A in rice, whose expression can be induced by salt and Cu stresses. In this study, three protein spots, identified as eIF5A (spot 32) and eIF5A-2 (spots 30 and 31), were down-regulated by excess Cu. It is unclear how reduced expression of eIF5A proteins under Cu toxicity affects Cu toxicity or tolerance in plants. Parker et al. [69] reported a significant down-regulation of eIF5A in rice after long-term salt stress treatment, which may be associated with premature senescence.

Cysteine proteinases (CysPs) are widely distributed in animals, microbes, and plants and play an important role in intracellular protein degradation. CysPs can be grouped into 15 families in five clans [70]. The papain-like proteases C1A (family C1, clan CA) are subdivided into cathepsin L-, B-, H- and F-like. In this study, three protein spots were identified as CysPs, including cathepsin B-like cysteine proteases (CBCP) (spot 10) and oryzain alpha chain flags precursor (spots 1 and 2), and both belong to the papain-proteases. These proteins were up-regulated in Cu-treated roots in both rice varieties. The up-regulation of CBCP (spot 10) was more pronounced in the Cu-tolerant variety B1139 than in the Cu-sensitive variety B1195. It was reported that CysPs participates in diverse processes: germination, senescence, abscission, programmed cell death and fruit ripening [6, 71, 72]. The expression of some CysPs may be induced in response to environmental stressors, such as cold, salt, drought, and wounding. To our knowledge, CysPs have not previously been identified as Cu-IMAC-binding proteins. Further studies are required to clarify the roles of CysPs in Cu-stressed plants.

Protein disulfide isomerase (PDI) is a thioredoxin superfamily oxidoreductase from the endoplasmic reticulum (ER). PDI catalyzes a wide range of thiol-disulfide exchange reactions and displays chaperone activity. Additionally, PDI is both a Ca- and a Cu-binding protein in animal cells [8, 9, 73]. Recently, PDI was also identified as a Cu-IMAC-binding protein in rice [14]. Chen et al. [74] reported that the heterologous expression of a PDI-like protein from *Methanothermobacter thermoautotrophicum* protected protein synthesis, increased protein stability, and enhanced Hg tolerance in rice. Exposure to a lower concentration of CuSO_4_ (< 20 μM) did not affect the expression of PDI in the marine alga *Ulva lactuca*, but exposure to a higher level significantly decreased the *PDI* transcription level [75]. In *Cucumis sativus* roots, one PDI was up-regulated under Fe deficiency, while another was down-regulated.

Ubiquitination is a well-characterized post-translational modification found in all eukaryotic cells. The conjugation of ubiquitin (Ub) to target proteins is sequentially carried out by E1 (Ub-activiting enzyme), E2 (Ub-conjugating enzyme, Ubc) and E3 (Ub-ligase). In this study, spot 35 was identified as ubiquitin-conjugating enzyme Spm2. The expression of this Cu-binding protein, like CSD protein (spot 34) and PR (spots 26), was seen in the Cu-tolerant variety B1139, but not in B1195. *Spm2* was isolated from *Schizosaccharomyces pombe* and is homologous to *methyl methanesulfonate sensitive 2* (*MMS2*), which encodes a Ub E2 enzyme variant (Uev). Uev is similar to E2 in both sequence and structure, but lacks the conserved active site cysteine residue and, thus, lacks conjugating activity on its own. It was shown that Uev promotes elysine-63-linked polyubiquitination and is involved in the DNA damage response [76]. The up-regulation of spm2 may promote error-free DNA replication and increase cell tolerance to Cu stress. Further studies are needed to elucidate the function of spm2 as a Cu-binding protein in Cu-stressed plants.

Spot 15 was identified as Epsilon-COP1, the Ɛ-subunit of coatomer protein I (COPI), which is up-regulated in B1139 and unchanged in B1195. COP I is a multimeric complex comprising seven submits: ɑ-, β-, β’-, γ-, δ-, ε-, and ζ-COP. The COPI coat is thought to be involved in transport between the Golgi cisternae, Golgi apparatus and endoplasmic reticulum (ER), and early and late endosomes and the endosomal compartment and the Golgi apparatus [77]. The up-regulation of Epsilon-COP1 may promote membrane traffic in plant cells under Cu stress.

CBP (spot 3) and CCoAOMT 1 (spot 14) levels were markedly increased by Cu stress in the Cu-tolerant variety B1139, but were unchanged in the Cu-sensitive variety B1195. CBPs are a large, heterogeneous group of proteins that participate in numerous cellular functions, such as control of cytosolic Ca^2+^ concentration, Ca^2+^ transport across the plasma membrane, or Ca^2+^-modulated sensors [78]. Cu^2+^ and Ca^2+^ ions share common sites and Cu^2+^ ions can replace Ca^2+^ ions bound to the C2A domain [79]. CCoAOMT catalyzes the methylation of caffeoyl-CoA to feruloyl-CoA and 5-hydroxyferuloyl-CoA to sinapoyl-CoA; thus it is thought to be involved in lignin biosynthesis. The expression level of CCoAOMT was shown to increase in several plants exposed to various abiotic and biotic stresses [14].

Two proteins were identified as adenosine kinase-like protein (ADK-L) (spot 13) and putative elongation factor 2 (EF-2) (spot 19), levels of which were markedly decreased by Cu stress in the Cu-sensitive variety B1195, but unchanged in the Cu-tolerant variety B1139. ADK catalyzes adenosine into AMP using one molecule of ATP and is a key player in the S-adenosyl-L-Met cycle, which provides methyl groups for a variety of transmethylation reactions. Further studies are needed to elucidate the functions of CBP and ADK in Cu-stressed plants. Elongation factors (EF1A, EF1B and EF-2) are fundamental regulatory proteins of the translational elongation step in higher plants, as in other eukaryotic organisms. EF-2 catalyzes the GTP-dependent translocation of peptidyl-tRNA from the A site to the P site of the ribosome during peptide chain elongation [80]. EF-2 contains the putative Cu-binding domain MX_7_H, one of the top six motifs reported by Kung et al. [10] from Arabidopsis roots, and CX_4_C, one of nine motifs reported by Smith et al. [9] from human liver cells. However, whether EF-2 proteins act in Cu homeostasis or toxicity through direct binding remains to be determined. Down-regulation of the EF-2 protein was also observed in Cd-treated *Phytolacca americana* [81] and B-deficient *Brassica napus*. A mutation in a gene encoding an EF-2-like protein (los1-1) in Arabidopsis blocks low-temperature-induced transcription of cold-responsive genes and reduces the capacity of plants to develop freezing tolerance [82].

Of the Cu-IMAC-binding proteins up-regulated by Cu-stress, we identified salt gene products (spots 7 and 27), salt stress-induced proteins (spot 9), putative glyoxylate induced proteins, and proteins containing dimeric α-β barrel domains. The up-regulation of salt stress-induced proteins was considerably greater than that of other proteins. However, their function in Cu-stressed plants is at present unknown.

## Conclusions

In this study, we investigated the differences in Cu-binding protein expression between Cu-tolerant and Cu-sensitive rice varieties using a new IMAC method. In total, 27 differentially expressed Cu-binding proteins were identified. Sixteen proteins were not previously identified as Cu-IMAC-binding proteins from plants or animals. These novel Cu-binding proteins were of four main types: proteins involved in antioxidant defense and detoxification, putative pathogenesis-related proteins, putative cold-shock domain proteins, and eukaryotic translation initiation factors. Besides, we also confirmed 29 protein spots significantly differentially expressed in two rice varieties under Cu stress (*P* < 0.05). Our research increases the understanding of the mechanisms involved in the heavy metal stress response and tolerance in plants. Further studies are required to clarify the roles of Cu ions in these putative Cu-binding proteins in plant cells, to determine if they are passive molecular targets of metal ions or active participants in metal tolerance.
